# Tracheobronchial Wall Thickening in a Patient With Inflammatory Bowel Disease

**DOI:** 10.1016/j.chpulm.2025.100186

**Published:** 2025-06-09

**Authors:** Jonathan Tse, Kevyn Ramos Laguna, Shuman Liu, Evan Yung, Chongiin Kim, Patrick Chan

**Affiliations:** a Department of Internal Medicine, Keck School of Medicine of USC, Los Angeles, CA; b Department of Pathology and Laboratory Medicine, Keck School of Medicine of USC, Los Angeles General Medical Center, Los Angeles, CA; c Division of Pulmonary and Critical Care, Los Angeles General Medical Center, Los Angeles, CA

## Abstract

A 43-year-old woman, born in Mexico, with a history of idiopathic thrombocytopenic purpura status after splenectomy and inflammatory bowel disease (IBD) favoring Crohn disease previously complicated by cytomegalovirus (CMV) colitis, presented with worsening abdominal and rectal pain. Over the past 6 months, she had 3 hospitalizations for biopsy-confirmed IBD flares, during which her immunosuppressive regimen was sequentially intensified from azathioprine and infliximab to high-dose corticosteroids and ultimately to upadacitinib for refractory disease.

Given recurrent symptoms, she underwent colonoscopy which demonstrated ulcerations throughout the colon and rectum, concerning for another inflammatory bowel disease (IBD) flare. She subsequently received a diverting loop ileostomy due to refractory disease. Biopsies later revealed evidence of cytomegalovirus (CMV) colitis, and she was started on ganciclovir. On hospitalization day 7, she became acutely hypoxic, desaturating to 85% on room air and requiring 5 L/min of oxygen via nasal cannula. Vital signs included a temperature of 38.3 °C, heart rate of 118 beats/min, BP of 117/78 mm Hg, and respiratory rate of 30 breaths/min. She reported feeling short of breath with throat pain and productive cough starting 3 days prior. At symptom onset, she was on ganciclovir, a prednisone taper starting at 45 mg, and had received upadacitinib 1 month prior. Physical examination was notable for a frail patient with coarse lower lobe lung sounds and mild expiratory wheezing. Initial blood work was significant for hemoglobin of 8.4 g/dL, platelets of 88 thousand cells per cubic millimeter (K/cumm), leukopenia of 2.6 K/cumm, erythrocyte sedimentation rate of 69 mm/h, C-reactive protein level of 117.5 mg/L, and procalcitonin level of 0.31 ng/mL. The acute change in respiratory status prompted a chest radiograph (Fig 1) and chest CT angiography with pulmonary artery protocol (Fig 2).

**Figure 1:**
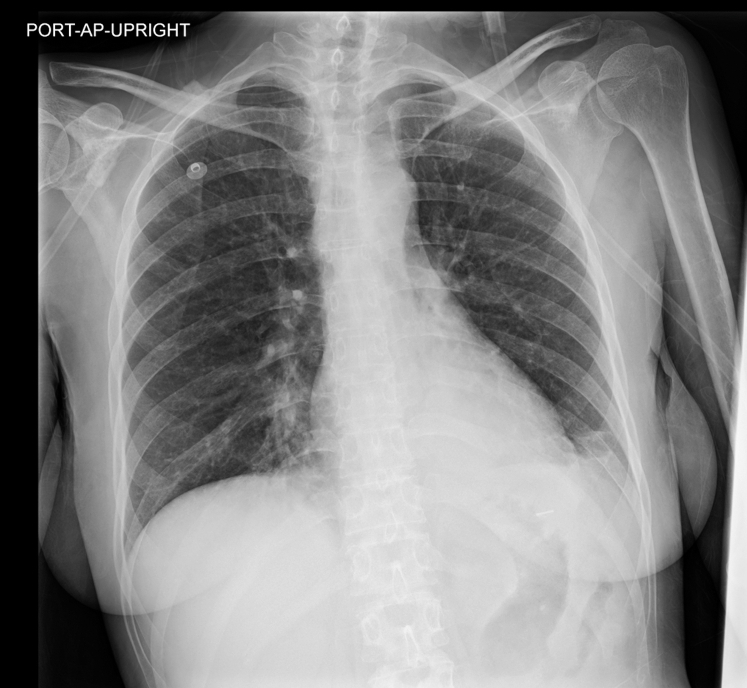
Upright anteroposterior chest radiograph demonstrating a medial opacity at the right lung base with larger consolidation in the retrocardiac region.

**Figure 2:**
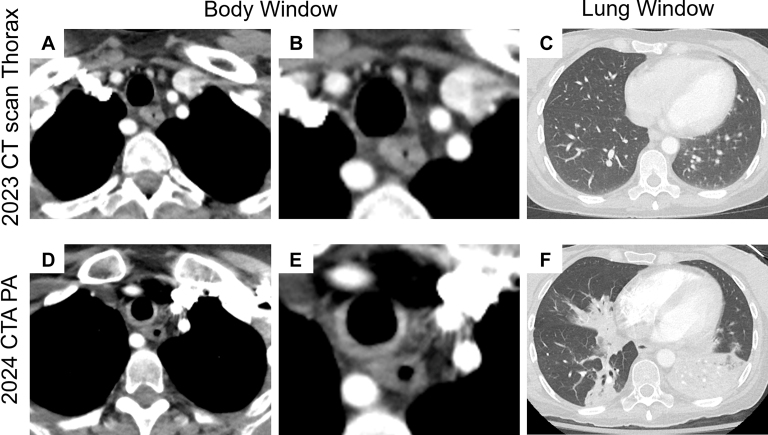
A-F, Chest CTA with pulmonary artery protocol on admission (D, E) demonstrating circumferential upper airway wall thickening and soft tissue density that extends along the bilateral proximal airways. Lung windows of the same scan show consolidative opacities most notable in the left lower lobe but also within the medial aspect of the right middle lobe and right lower lobe (F). CT thorax scan from 1 y before admission provided for reference with corresponding cuts (A-C). CTA = CT angiography; PA = pulmonary artery.

Subsequently, a broad bacterial, viral, and fungal workup was sent. Notable positive results included a lactate dehydrogenase of 324 units/L, serum CMV DNA polymerase chain reaction of 17,076 International Units/mL, and a positive serum 1-3-β-d-glucan of 190 pg/mL. Notable negative results included blood culture, sputum cultures, communicable respiratory TB evaluation, HIV screen, urine *Histoplasma* screen, serum *Blastomyces* antibody, serum *Cryptococcal* antigen, and serum *Aspergillus* antigen enzyme immunoassay. Although the serum studies were pending, a flexible bronchoscopy (Fig 3) was performed on hospitalization day 9 given her concerning CT findings.

**Figure 3:**
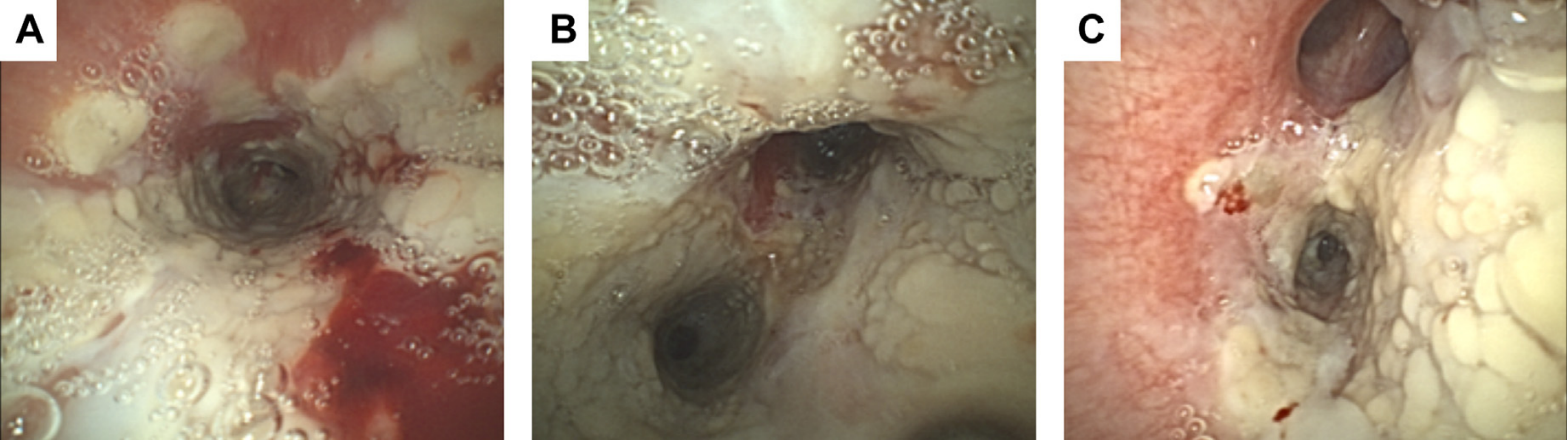
A-C, Flexible bronchoscopy was used to examine at least to the first bilateral subsegmental level. On initial bronchoscopy, diffuse white plaques were noted from the proximal trachea (A), carina (B), and right mainstem bronchus (C). The airway mucosa was noted to be friable.

Given these bronchoscopy and CT findings in the setting of severe immunosuppression, there was high suspicion for infection. The decision was made to initiate therapy and rapidly taper off immunosuppression, despite repeated hospitalizations for IBD flares. The patient’s hospital course was complicated by worsening pancytopenia, thought to be secondary to ganciclovir. Filgrastim was initiated for drug-related bone marrow suppression. Her respiratory status improved after the rapid taper of steroids and initiation of appropriate therapy, eventually progressing to room air by hospitalization day 15. Left lower lobe bronchoalveolar lavage (BAL) cytology and pathology from endobronchial biopsy of the right upper lobe (Fig 4) performed on hospital day 17 confirmed our diagnosis.

**Figure 4:**
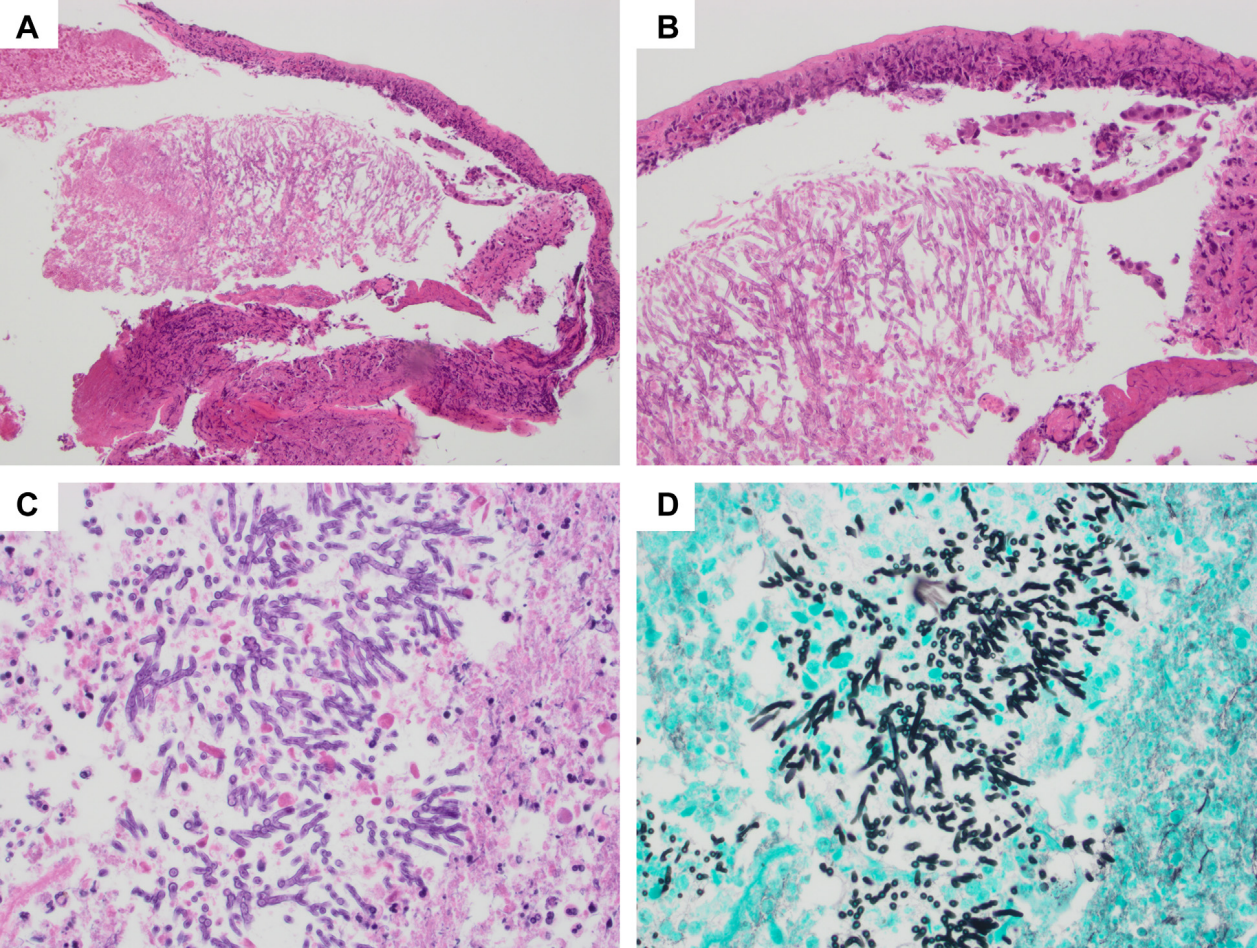
A-D, Photomicrographs of the patient’s pathology specimens. Low and mid-magnification photomicrographs of the endobronchial biopsy showing fungal hyphae adjacent to fibrinopurulent debris and epithelium with marked reactive changes (A, hematoxylin and eosin [H&E] ×100; B, H&E ×200). High magnification photomicrographs of the bronchoalveolar lavage showing fungal elements intermixed with abundant necroinflammatory exudate (C, H&E ×400). These fungal elements are highlighted with a Grocott-Gomori methenamine silver stain (D, ×400).

*What is the diagnosis?*

*Diagnosis:* Invasive tracheobronchial aspergillosis (pseudomembranous tracheobronchial aspergillosis)

## Discussion

### Radiologic Discussion

Compared with an unremarkable CT scan done 1 year prior, the patient’s CT scan (Fig 2) obtained after her new respiratory symptoms demonstrated interval development of circumferential upper airway wall thickening. The radiologists noted abnormal soft tissue density that extended along the bilateral proximal airways. This transitioned to consolidative opacities most notable in the left lower lobe and right lower lung fields. Multiple airway-centric nodular ground-glass opacities were noted in the bilateral upper lobes. Our focus became determining whether the tracheobronchial thickening represented IBD-associated airway disease or an opportunistic infection. This proved to be challenging from radiographic imaging alone. Many of these findings can be seen in IBD airway disease, which may develop or worsen after total colectomy.^1^ These radiographic findings were ultimately reported as being consistent with the airway manifestations of IBD, but unable to exclude an infectious component.

Invasive tracheobronchial aspergillosis (ITBA) can pose a diagnostic challenge. The chest radiograph for the patient had nonspecific bibasilar opacities. CT scans, although also often nonspecific, can reveal subtle findings that, in the appropriate clinical context, may suggest an underlying infectious process. Early stage ITBA can present with tracheal or bronchial wall thickening, whereas later-stage disease can present with peribronchial consolidations, multiple ill-defined centrilobular nodules, and cavitations.^2^ However, in 1 case series of 12 patients, only 2 patients demonstrated the aforementioned radiologic findings.^3^ Given the recent reactivation of CMV in the setting of immunosuppression, we were highly concerned for additional opportunistic infections. This prompted further evaluation and pursuit of diagnostic testing with bronchoscopy.

### Pathologic Discussion

Serologic testing and even culture data may yield negative results, as seen in this case. Although certain tests can be helpful if positive in guiding diagnosis, these tests often have limited sensitivity. Thus, the diagnosis of ITBA and its differentiation into its subtype, *Aspergillus* tracheobronchitis, ulcerative *Aspergillus* tracheobronchitis, and pseudomembranous *Aspergillus* tracheobronchitis (PMATB), can only be made via visualization of the fungus and the affected tissue.^4^ As such, bronchoscopy with microscopy and culture of collected samples is essential for making the diagnosis. During bronchoscopy, given the concern for opportunistic infections and IBD disease presenting as a flare, a biopsy was performed. The BAL sample demonstrated findings consistent with *Aspergillus* on cytology, was positive for *Aspergillus galactomannan* on BAL enzyme immunoassay with an index value of 5.49, and eventually grew *Aspergillus fumigatus* on fungal cultures. PMATB, the most invasive form of ITBA and the subtype this patient presented with, is identified by the visualization of pseudomembranes composed of sloughing necrotic tissue and mucus.^4^ These pseudomembranes were visualized in the patient diffusely in the trachea and into both the right and left bronchial tree. Endobronchial biopsy revealed fungal hyphae adjacent to fibrinopurulent debris and epithelium with marked reactive changes. The BAL specimen demonstrated fungal elements within necrotic tissue. Considering these findings, a diagnosis of PMATB was established.

### Clinical Discussion

The tracheobronchial findings on CT scan prompted a broad differential, considering the patient’s history and immunocompromised status. Given recurrent admissions for IBD flares and the known potential of IBD to involve the airways, IBD-related airway involvement was a leading consideration. However, the patient’s immunosuppression raised the likelihood of infection, with potential causes including TB, fungal infections, bacterial pathogens, and even viral infections, especially with her known active CMV infection. Despite no personal history of malignancy, metastatic malignancy was considered due to the association between IBD and malignancy. Classic inflammatory conditions (eg, tracheopathia osteochondroplastica, amyloidosis) were deemed less likely in the context of the aforementioned differentials.

Although pseudomembranous tracheobronchitis is rare, *Aspergillus* species remains overwhelmingly the most common etiology, as seen in this patient. Nonetheless, the condition can arise from a broad range of organisms.^5^ *Coccidioides* species and mucormycosis are 2 other cited fungal etiologies, with rarer reports of *Candida* species, *Histoplasma* species, and *Cryptococcus neoformans*. Among bacterial etiologies, *Staphylococcus* species is the most common; however, *Streptococcus* species, *Pseudomonas* species, *Mycoplasma* species, and *Corynebacterium* species have also been reported. Viral tracheobronchitis appears to be much less recognized, but there are reported cases of influenza, herpes, and parainfluenza infections. In almost all cases of viral tracheobronchitis, the patients demonstrated preexisting immunocompromised conditions or had concurrent bacterial etiologies.

Pulmonary aspergillosis has many manifestations, most commonly involving the parenchyma, ranging from relatively benign aspergilloma to invasive disease. In rare cases, invasive pulmonary aspergillosis demonstrates severe invasion of the tracheal and bronchial tissues in what is called ITBA.^4^ ITBA is often seen in lung transplant recipients and patients with hematologic malignancies with or without stem cell transplants.^6^^,^^7^ These patients are more susceptible to infection due to their highly immunocompromised state. Mortality can be as high as 72%.^8^ Although not well understood, there appears to be a difference in the clinical course and prognosis of these patients depending on the etiology of immunosuppression, with lung transplant recipients having better prognosis than patients with hematologic malignancy.^4^ One thought is that immunosuppression can be readily modified in a transplant patient by decreasing immunosuppressive medications, whereas patients with hematologic malignancies remain immunosuppressed due to their underlying malignancy. Additionally, ITAB and PMATB have been documented in patients demonstrating other severe systemic immunosuppressive risk factors including HIV/AIDS, solid organ transplants other than lung, other malignancies, antineoplastic treatment, neutropenia, and systemic corticosteroid use.^4^ Similarly, this patient likely developed PMATB secondary to her immunosuppression for her Crohn disease.

**Figure 5:**
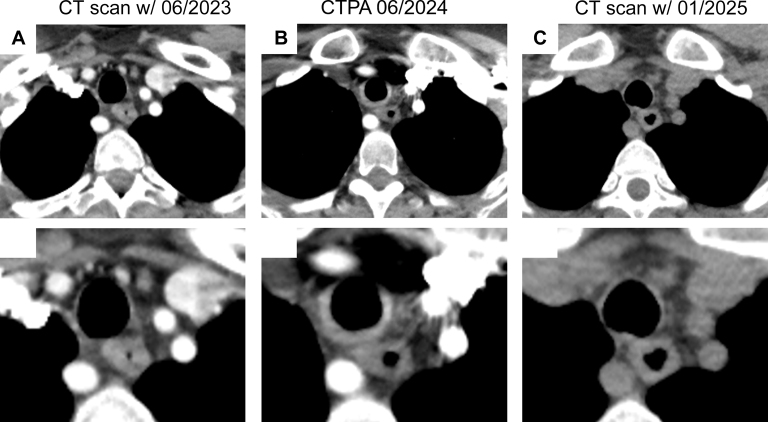
A-C, When compared with the CT thorax scan from 1 y prior (A), the chest CTA with pulmonary artery protocol (B) at the time of admission demonstrated circumferential upper airway wall thickening along the proximal airway. After 6 mo of antifungal treatment, the upper airway thickening resolved on repeat imaging (C). CTA = CT angiography; PA = pulmonary artery; w/ = with; w/o = without.

**Figure 6:**
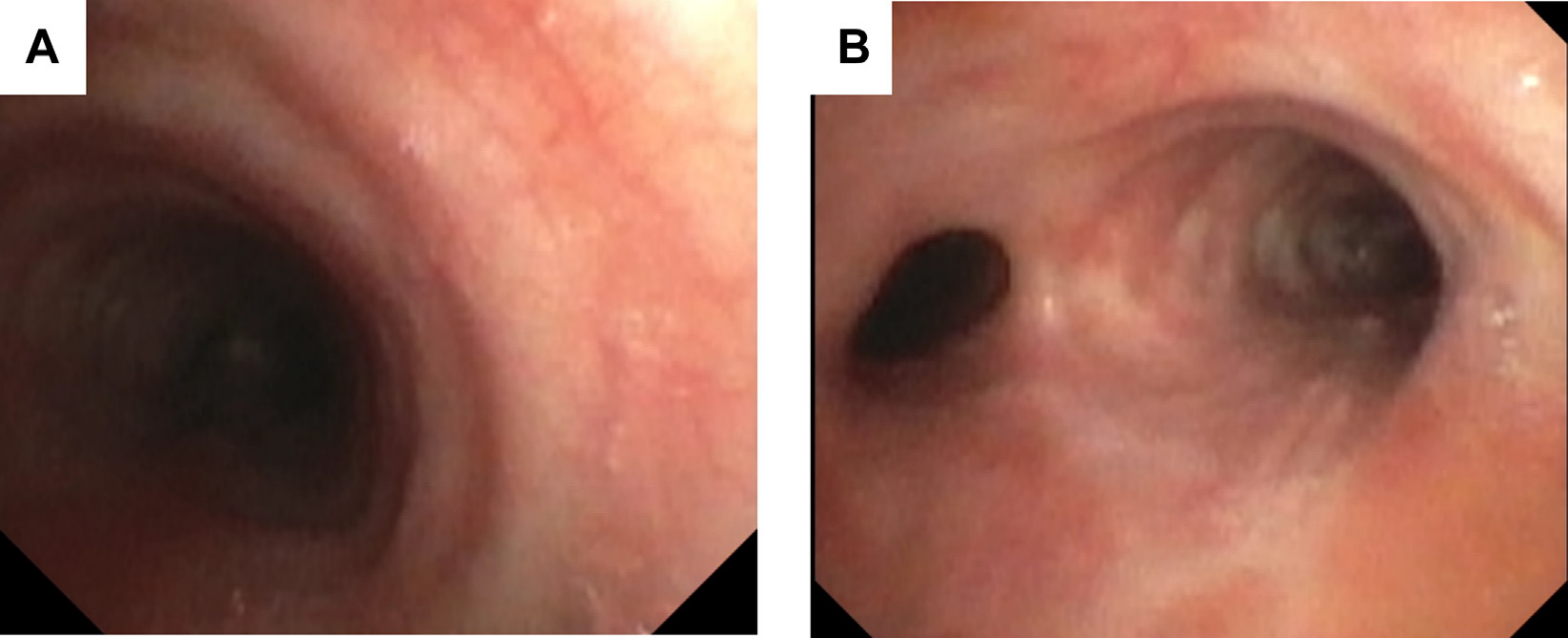
A, B, Repeat bronchoscopy after 3 mo of treatment demonstrated no plaques in the proximal trachea (A) and minimal residual plaques in the distal trachea (B).

Studies have demonstrated that voriconazole is more effective and better tolerated than amphotericin B. Voriconazole is also more efficacious than salvage therapy via other antifungal therapies including liposomal amphotericin B, itraconazole, and caspofungin.^9^^,^^10^ As such, this patient was started on voriconazole and tapered off her immunosuppression completely. She did end up transferring to the ICU due to tachypnea and respiratory status; however, she improved and was eventually discharged. She had a repeat CT thorax scan and bronchoscopy as an outpatient, which demonstrated resolution of tracheal wall thickening (Fig 5) and significant improvement in her tracheobronchial disease (Fig 6). She remains on antifungal therapy for a planned minimum course of 3 months with plans for serial imaging and possibly repeat bronchoscopy as an outpatient. Her duration of treatment will depend on her response and her need for immunosuppression. She has close outpatient follow-up with pulmonology, infectious disease, gastroenterology, and colorectal surgery for multidisciplinary management of her complicated IBD and opportunistic infections.

## Conclusion

• ITBA may be difficult to diagnose given its rarity, nonspecific presentation, and lack of reliable diagnostic testing.
• Bronchoscopy with microscopy and culture is currently the only definitive modality of diagnosing PMATB.
• Clinicians should have a high clinical suspicion for PMATB in immunocompromised patients with tracheobronchial thickening noted on CT imaging.
• Early diagnosis and timely treatment of ITBA is paramount and can result in favorable outcomes.

## Financial/Nonfinancial Disclosures

None declared.
